# High‐resolution distribution modeling of a threatened short‐range endemic plant informed by edaphic factors

**DOI:** 10.1002/ece3.5933

**Published:** 2019-12-15

**Authors:** Sean Tomlinson, Wolfgang Lewandrowski, Carole P. Elliott, Ben P. Miller, Shane R. Turner

**Affiliations:** ^1^ School of Molecular & Life Sciences Curtin University Perth WA Australia; ^2^ Kings Park Science Department of Biodiversity, Conservation and Attractions West Perth WA Australia; ^3^ School of Biological Sciences University of Western Australia Crawley WA Australia

**Keywords:** banded ironstone formation, conservation biology, rare species, *Ricinocarpos brevis*, soil water potential, species distribution modeling

## Abstract

1. Short‐range endemic plants often have edaphic specializations that, with their restricted distributions, expose them to increased risk of anthropogenic extinction.
2. Here, we present a modeling approach to understand habitat suitability for *Ricinocarpos brevis* R.J.F.Hend. & Mollemans (Euphorbiaceae), a threatened shrub confined to three isolated populations in the semi‐arid south‐west of Western Australia. The model is a maximum entropy species distribution projection constructed on the basis of physical soil characteristics and geomorphology data at approximately 25 m^2^ (1 arc‐second) resolution.
3. The model predicts the species to occur on shallow, low bulk density soils that are located high in the landscape. The model shows high affinity (72.1% average likelihood of occurrence) for the known populations of *R. brevis*, as well as identifying likely locations that are not currently known to support the species. There was a strong relationship between the likelihood of *R. brevis* occurrence and soil moisture content that the model estimated at a depth of 20 cm.
4. We advocate that our approach should be standardized using publicly available data to generate testable hypotheses for the distribution and conservation management of short‐range endemic plant species for all of continental Australia.

## Graphical abstract

A novel workflow was developed to produce species distribution models at extremely high (1 s) resolution for a range‐restricted species. The modeling data were also able to identify microclimatic factors associated with high likelihood of occurrence.

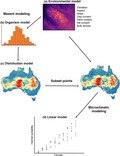

## 1 INTRODUCTION

Species rarity is a common state, driven by diverse factors, that manifests in different ways (Fiedler & Ahouse, 1992; Kruckeberg & Rabinowitz, 1985). One form of rarity involves the localization of a species to a relatively small distribution and often an association with specialized habitat features (Lavergne, Thompson, Garnier, & Debussche, 2004), a pattern termed short‐range endemism (SRE; Sreenivasulu and Amritphale, 1999). Short‐range endemism may not be necessarily associated with either numerical rarity, since large and stable populations can occur in these specialized habitats, nor with high extinction risk (Murray, Thrall, Gill, & Nicotra, 2002), even throughout periods of substantial climatic changes (Byrne et al., 2018; Patsiou, Conti, Zimmermann, Theordoris, & Randin, 2014). However, it does increase susceptibility to anthropogenic extirpation where those habitats might be the target of intense habitat modification (Burgman, Keith, Hopper, Widyatmoko, & Drill, 2007; Coates et al., 2014; Gibson, Coates, Leeuwen, & Yates, 2015). Conservation of these species is challenging in situations where their only known habitats are proposed to be either removed or significantly altered (Lande, Landweberg, & Dobson, 1999).

Short‐range endemism is an intriguing phenomenon because it presents peculiar patterns that do not intuitively meet with our expectations of adaptation and evolution. Quite often the landscape presents large areas of apparently suitable habitat in which SRE species are not present and appear never to have been present for no obvious reason/s (Robinson, Virgilio, Temple‐Smith, Hesford, & Wardell‐Johnson, 2018). In the event that these habitats are separated by some potential dispersal or geographic barriers, however small, stochastic local extinction with a failure to reinvade is often invoked as the reason for this patchiness (Fiedler & Ahouse, 1992; Hopper & Gioia, 2004). This suggestion however, is not always a feasible explanation in apparently contiguous habitats. In these circumstances, it is often presumed that there are cryptic environmental conditions that either provide a competitive advantage to SRE species by excluding competitors, or provide a specific requirement that is not available anywhere else in the landscape (Lavergne et al., 2004). While these specific requirements are critical to defining the distributions of the species naturally, they also place constraints on many of the activities typically used to conserve species like this, such as the identification of unknown populations, the establishment of insurance populations, or translocation to new habitats should the existing populations come under threat (Armstrong & Seddon, 2008; Maschinski & Albrecht, 2017).

Ecological management requires quantification of threatening processes and constraints (e.g., Alagador, Cerdeira, & Araújo, 2015) as well as understanding species biology and spatial ecology (Fiedler & Ahouse, 1992). Correlative distribution modeling can be implemented from basic, and sometimes freely available, data to offer initial insights into species‐specific niche constraints (Kearney & Porter, 2009). In an applied context, correlative distribution modeling has been used to guide translocations and assisted colonizations (Ferrarini et al., 2016; Regan et al., 2012). While climate is typically used as the basis for such modeling, available global climate projections (e.g., Hijmans, Cameron, Parra, Jones, & Jarvis, 2005; Kearney, Isaac, & Porter, 2014; Karger et al., 2017) generally produced at 30 arc‐seconds (i.e., >0.5 km^2^) or more resolution, cannot capture specific nuances of microclimatic variability critical to the conservation management of short‐range (<0.1 km^2^) endemic species. Climatically informed models may thus fail to model distribution of SRE species and require alternative modeling approaches (Beauregard & de Blois, 2014; Thuiller, 2013). However, interpolating large‐scale atmospheric conditions to estimate atmospheric temperature and rainfall patterns at higher resolution by “quasi‐mechanistic statistical downscaling” (Karger et al., 2017; Xu & Hutchinson, 2013) may potentially be highly informative to project‐specific species distribution models (SDMs). Outputs include air temperature, relative humidity, and wind speed, solar radiation, predicted precipitation, and soil temperature, and moisture content at finer resolution than global or regional climate data (Kearney & Porter, 2016). These latter variables of soil microhabitat are key drivers of many physiological processes in plants (Lambers, Chapin, & Pons, 2008). Thus, they are of key interest in modeling plant distributions, particularly at the resolution required to identify habitats for SRE taxa, where climatic conditions might be very similar across a landscape, but soil microhabitats can change cryptically over short distances. Understanding how below‐ground conditions can change even when above‐ground conditions appear homogeneous might provide substantial insights into ecological constraints defining the current distribution of SRE species and thereby inform their conservation and restoration.

**Figure 1:**
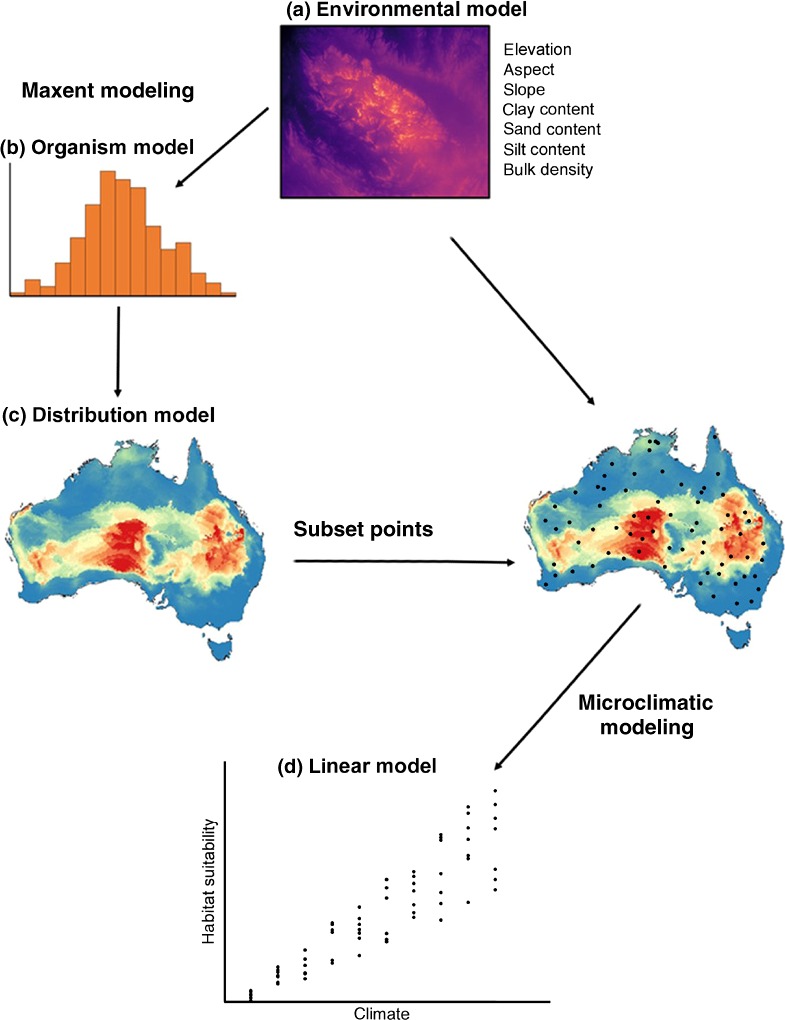
Our model workflow used a series of topographic and edaphic elements to define the environmental layers (a). Through MaxEnt (Phillips & Dudik, 2008), we developed an organism model (b) using locations of known species occurrence to project our final species distribution model (c). To interrogate the microclimatic correlates of high habitat suitability, we developed a microclimatic model for 2,000 subset locations across our study area using the micro_global algorithm in NicheMapR (Kearney & Porter, 2016) and then used a linear modeling approach (d) to seek correlation between microclimatic conditions and high habitat suitability. This workflow substantially reduces the computing power required by reducing the microclimatic calculations to a smaller subset of locations within the projection region

The challenge for conservation agencies or other stakeholders in applying microclimatic data at appropriate scale and resolution is the requirement for substantial computational power to develop high‐resolution environmental surfaces (Buckley et al., 2010). The problem of project‐specific spatial data resolution has been approached in previous SDMs (e.g., Keppel et al., 2017) by training models using high‐resolution topographic data collected from aerial photography, photogrammetry, and Lidar scanning, proving capable of resolving plausible model projections. The interpretation of these models is somewhat limited because topographical elements do not relate directly to biological processes that constrain distributions (Buermann et al., 2008; He et al., 2015). At best, they are proxies of biologically relevant microclimatic forcing factors, and, as such, offer limited insight into the ecological requirements of the organism. We propose a workflow designed to minimize this heavy computational requirement (Figure 1) and potentially identify more directly biologically meaningful models. This workflow establishes SDMs on the basis of standardized data describing edaphic characteristics, and then further interrogates microclimatic correlates of habitat suitability in a subset of locations. As such, it may prove practical to develop high‐resolution SDMs for SRE species independent of climate using these data layers. Interpreting the influences of microclimatic correlates can then be pursued by training a microclimatic algorithm on a subset of the edaphic data. In this quasi‐mechanistic manner, patterns of endemism may be interrogated to either determine locations of potentially unidentified populations of SRE species, or to inform the establishment of new insurance populations or mitigation‐driven translocations.

Banded ironstone formations (BIF) are known for their high floral endemism, and their vulnerability to anthropogenic disturbance through mining and provide a natural testbed for developing the SDMs discussed above. One such example is the threatened SRE shrub, *Ricinocarpos brevis* R.J.F.Hend. & Mollemans (Euphorbiaceae), which is recognized under Australian federal legislation (the *Environment Protection and Biodiversity Conservation Act 1999*) and confined to isolated locations in the semi‐arid south‐west of Western Australia (Krauss & Anthony, 2018). Populations comprise of ~16,000 individuals over a total area of 2,000 km^2^, of which only 8 km^2^ is specifically occupied (Department of Environment & Conservation, 2011). Focussing on *R. brevis* to develop a standardized SDM approach presented several advantages over other SRE flora. For example, there are multiple, largely intact populations, all of which are restricted to very small spatial extents at different locations. The species has no sympatric close relatives over its area of known occupancy, thus eliminating the identification of potential habitats already filled by congenerics from our interpretation. Further, the species is associated with only a few landforms and vegetation communities which are more widely dispersed than the known populations of *R. brevis*. Consequently, we aim to demonstrate the practical value of our new workflow on a SRE species with clear conservation significance and for which efforts at in situ restoration are already underway (Gibson, Coates, & Thiele, 2007; Gibson, Yates, & Dillon, 2010; Krauss & Anthony, 2018; Turner et al., 2018).

We hypothesized that the SDM of *R. brevis* could be created on the basis of edaphic constraints and that potentially suitable habitat within the wider landscape remains unused by this species and therefore, may be targeted in future translocations. As such, the high‐resolution SDM reported here had the following objectives: identifying landscape drivers of the distribution of *R. brevis*, and, by extrapolating the model across a larger region, identifying locations outside of the current distribution that, according to the model, would be most likely to support populations of *R. brevis*. Here we explore the increased modeling flexibility and interpretation possible by training a correlative distribution model using ultra‐high‐resolution data describing geomorphology and soils that can also inform the calculation of microclimatic factors. This was undertaken to identify the microclimatic and ecophysiological correlates of rarity in a BIF endemic species in Western Australia, a landscape noted for its high rates of short‐range endemism and economically valuable iron ore.

## 2 MATERIALS AND METHODS

### 2.1 Data sources

*Ricinocarpos brevis* has been heavily surveyed by the mineral extraction industry under their obligations to the Australian government to develop management plans to offset the impacts on the species resulting from mining. Collectively, 13,081 individuals are known across three populations, and their locations have all been recorded. We used these locations as our training data, ultimately contributing 762 point locations after local duplicates had been removed (see Section 2.2) to construct two species distribution models for *R. brevis*. The SDM that used edaphic and geomorphological input data only is referred to as the edaphic‐SDM, and the SDM that used climate input data only is referred to as the climate‐SDM below.

**Figure 2:**
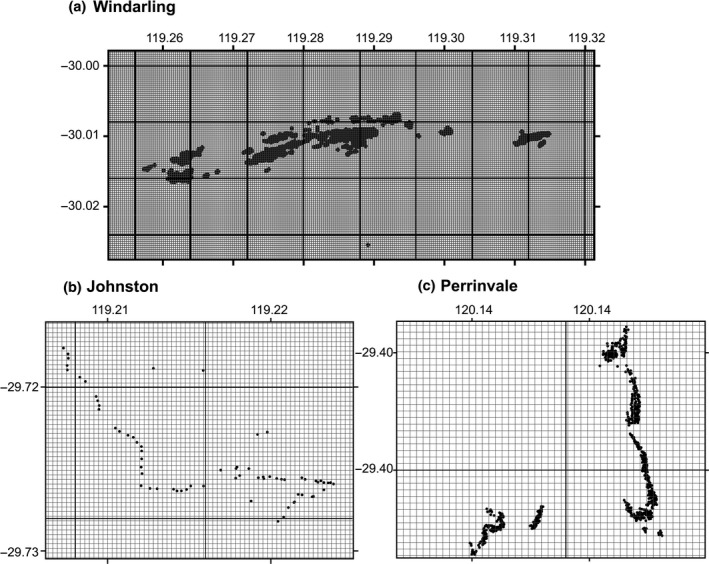
A comparison of different resolutions of available data for species distribution modeling. The black grid represents the scale of the majority of freely available climate layers at 30 arc‐second resolution. At the location where this study took place, that translates to a 754 m grid size. The gray grid represents the resolution of the layers that we used for our modeling at 1 arc‐second (roughly 25 m grid size). As can be seen, the coarser resolution encompasses the known locations (black points) in a small number of grid squares, reducing the training power substantially. Furthermore, the resulting models only project at the highest resolution of environmental layers available, which limits the management insights and guidance available. This is of substantial importance when trying to guide the management or translocation of a short‐range endemic species such as *Ricinocarpos brevis*, shown here, where the entire know distribution (roughly 8 km^2^) falls on three ridges, encompassed by 17 30 arc‐second grids

**Figure 3:**
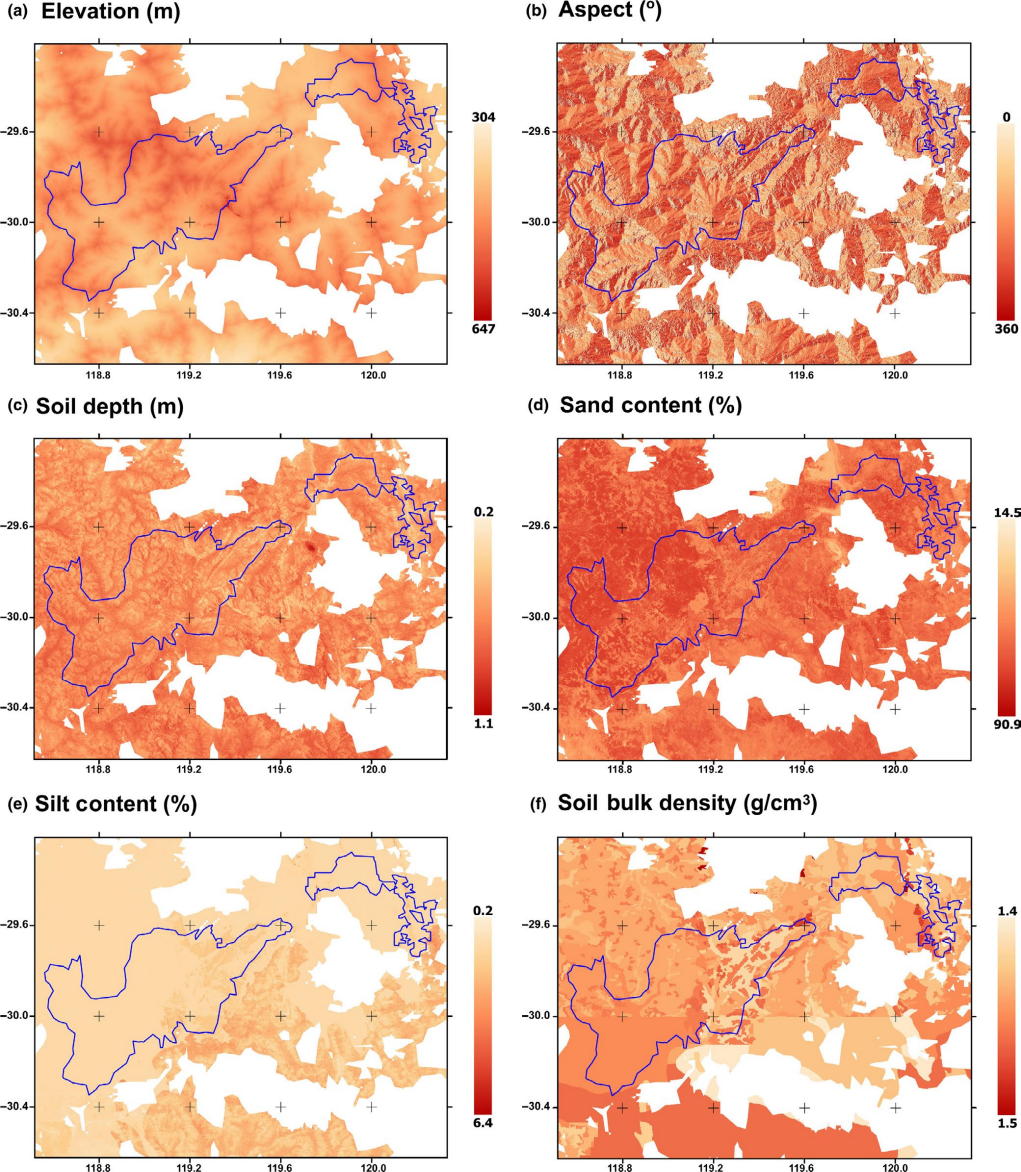
Spatial data used to train the MaxEnt distribution projections. (a) Elevation; (b) Aspect derived from Gallant et al. (2011), and Gallant and Austin (2012a, 2012b; (c) Soil depth; (d) Soil sand content; (e) Soil silt content extracted from Viscarra Rossel et al. (2014a, 2014b, 2014c); and (f) Soil bulk density provided by the Australian Collaborative Land Evaluation Program ACLEP. The polygon indicates the training region for the modeling process

The edaphic‐SDM presented was constructed around publicly available data sets describing geomorphology and physical soil characteristics at one arc‐second resolution (approximately 25 m^2^) or higher, providing the higher resolution required to model populations over small geographic ranges (Figure 2). Ultra‐high‐resolution digital elevation, aspect, and slope data were extracted from Gallant et al. (2011), and Gallant and Austin (2012a, 2012b), respectively. Soil properties including clay, sand, and silt percentage at 15 cm depth were extracted from Viscarra Rossel et al. (2014a); Viscarra Rossel et al. (2014b); Viscarra Rossel et al. (2014b). Soil bulk density (Mg/m^3^) and depth were interpolated for each 25 m^2^ grid location from national soil data provided by the Australian Collaborative Land Evaluation Program ACLEP, endorsed through the National Committee on Soil and Terrain NCST (http://www.clw.csiro.au/aclep). The spatial extent of the training data set (Elith et al., 2011; Van der Wal, Schoo, Graham, & Williams, 2009) was objectively defined following Webber, Yates, et al. (2011) by spatially intersecting the distribution records for all known locations of *R. brevis* with a continental environmental domain classification (Mackey, Berry, & Brown, 2008). The training region was defined by selecting the environmental domain polygons that encompass the known occurrence locations of the three populations at the Perrinvale, Johnston, and Windarling Ranges, (Figure 3), with neighboring polygons and “second neighbor” polygons also included. To provide a comparison of our non‐climatically informed SDM to a more traditional approach, we calculated a down‐scaled microclimatic model at 1 arc‐sec resolution using the “micro_global” algorithm of the *NicheMapR* package (Kearney, 2016) in *R* (R Core Team, 2016). The details of this process are more completely explained below. We constructed our climate‐SDM for three landscapes encompassing a 10 km buffer around all the known individuals of *R. brevis* that described patterns in air temperature, solar irradiance, soil water potential at a depth of 20 cm and soil temperature at a depth of 10 cm during the reproductive season of the species in the austral winter. These microclimatic parameters correspond to the parameters Bio08, Bio24, and Bio32 of the established “bioclim” data set (Kriticos, Jarošik, & Ota, 2014; Xu & Hutchinson, 2013). Soil temperatures are not generally included in the established global climate layers.

### 2.2 Species distribution modeling

**Figure 4:**
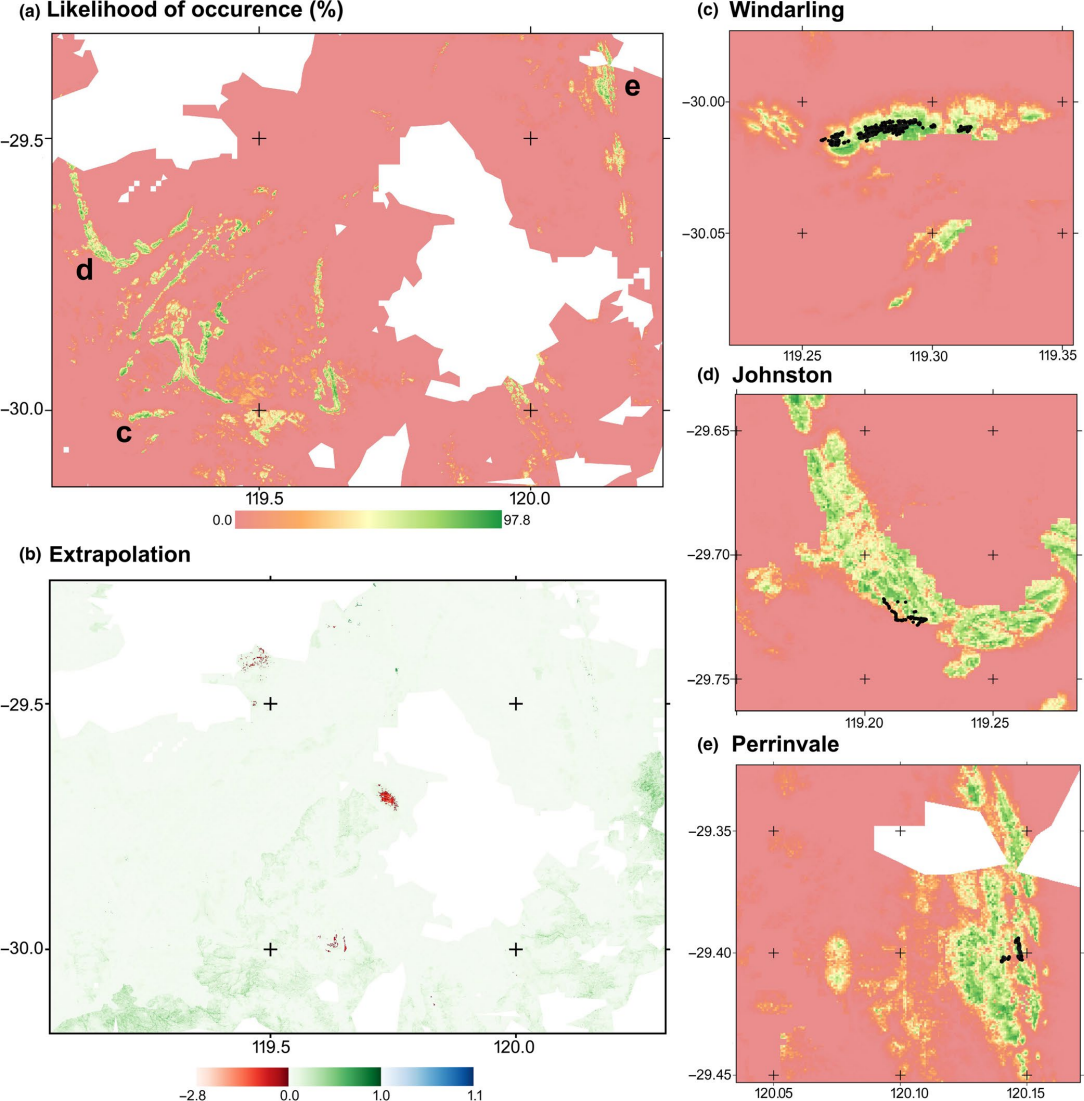
(a) Projected species distribution of *R. brevis* likelihood of occurrence derived from the Maxent model. Increasing intensity of color (from red to green) indicates a high likelihood of occurrence. (b) Comparison of covariate space in the projection region, compared with the conditions encompassed within the training region calculated based on the Mahalanobis distance. Following the thematic scheme of Mesgaran et al. (2014), areas in red have one or more environmental variables outside the range present in the training data, while areas in blue have covariate interactions that are outside the range of those present in the training data, and so represent spatial range where the model is extrapolating, and require more cautious interpretation than green areas, which fall within the bounds of the training region. The remaining three panels represent projected likelihood of occurrence at Windarling Range (c), Johnston Range (d), and Perrinvale Range (e)

We used the maximum entropy algorithm implemented in MaxEnt version 3.3.3a (Phillips, Anderson, & Schapire, 2006; Phillips & Dudik, 2008) to model the local distribution of *R. brevis* within the three known populations. Default MaxEnt parameter settings (maximum number of background points 10,000; regularization multiplier 1; auto features; maximum iterations 500; and convergence threshold 0.00001 and duplicate records deleted) were used to develop logistic likelihoods of occurrence, ranging from zero at the lowest likelihood of presence to one at the strongest prediction for presence (Phillips, 2008). In applying the 10th percentile training presence, which omits the 10% most extreme presence observations, we sought to more accurately represent the “core of the species' present range” (Morueta‐Holme, Fløjgaard, & Svenning, 2010). We then used a 10% test presence, which reserves 10% of the known occurrence locations for testing the resulting models (Phillips et al., 2006; Phillips & Dudik, 2008). Pilot edaphic‐SDMs were developed using all the available candidate layers (elevation, aspect, slope, clay, sand and silt content, and bulk density) and refined by removing layers that contributed less than 5% contribution to fit. The final model was constructed using only elevation, aspect, soil depth, sand and silt content, and bulk density (Figure 3) and projected to a 50,000 km^2^ area that included the entirety of the Windarling, Perrinvale and Johnston Ranges, and much of the surrounding plains and other intervening landforms (Figure 4). To explore patterns of extrapolation in the resulting model projection, we measured similarity of the covariance matrix between training and projection locations with Mahalanobis distance (Mesgaran, Cousens, & Webber, 2014) using the *ecospat* package (Di Cola et al., 2017) in the *R* statistical environment (R Core Team, 2016) to compare the model backgrounds with the projection to the wider project area.

### 2.3 Microclimatic Interpretations

Geomorphological and soil characteristics used in our edaphic‐SDM were chosen for their capacity to parameterize a microclimatic model using the “micro_global” algorithm of the *NicheMapR* package (Kearney, 2016) in *R* (R Core Team, 2016). This algorithm, developed to provide context for mechanistic distribution modeling (Kearney & Porter, 2016), interpolates a climate model (New, Lister, Hulme, & Makin, 2002) to estimate both atmospheric and substrate conditions associated with areas predicted by the SDM. By incorporating elevation, slope, aspect, and soil texture, these calculations can be modified as a function of shading and soil physical properties (Kearney, Isaac, et al., 2014; Kearney, Shamakhy, et al., 2014). Substrate conditions, to our knowledge, are not well‐captured by other existing climate models (e.g., Hijmans et al., 2005; Karger et al., 2017), despite their critical value in estimating the abiotic niche. Two thousand random points were selected across the training range, and their physical soil characteristics were summarized into a format appropriate for *NicheMapR* following a freely available soil texture calculator produced by the United States Department of Agriculture (https://www.nrcs.usda.gov/wps/portal/nrcs/detail/soils/survey/?cxml:id=nrcs142p2_054167), adapted to a computer algorithm similar to Gerakis and Baer (1999). For lack of any quantified proxies for vegetation shading, all microclimatic projections were run assuming full sun, with the recognition that this will overestimate temperature properties and underestimate soil water potentials under vegetation and associated leaf litter. The model projections were averaged following calculation of microclimatic conditions every hour over a 10‐year projection period. The resulting microclimatic projections included average annual air temperature at a reference height of 1.0 m (corresponding to typical *R. brevis* canopy height), soil temperatures and water potentials at 0, 0.2 and 1.0 m depth, and annual average solar radiation (i.e., hours of sunlight). These three soil depths capture the biologically active layer of topsoil (Berrigan & Partridge, 1997; Jasper, Robson, & Abbott, 1988), relevant for seed germination, seedling establishment, plant root growth, and water acquisition, together with root zone conditions.

Although these are less completely resolved microclimatic correlates than published data sets, which dissect the raw climatic data according to bioclimatic relevance (Hijmans et al., 2005; Kearney, Isaac, et al., 2014; Kriticos et al., 2012), they represent the simplest set of variables known to have physiological significance to plant performance. In order to interpret the ecophysiological correlates of modeled occurrence likelihood, a linear model was constructed, comparing likelihood of occurrence against all the microclimatic projections in *R*. A model averaging approach was carried out in the *MuMIn* package (Barton, 2013) using Akaike Information Criterion values (AICc, corrected for small sample bias; Burnham and Anderson, 2002) to find the most parsimonious model linking likelihood of occurrence with microclimatic parameters.

**Figure 5:**
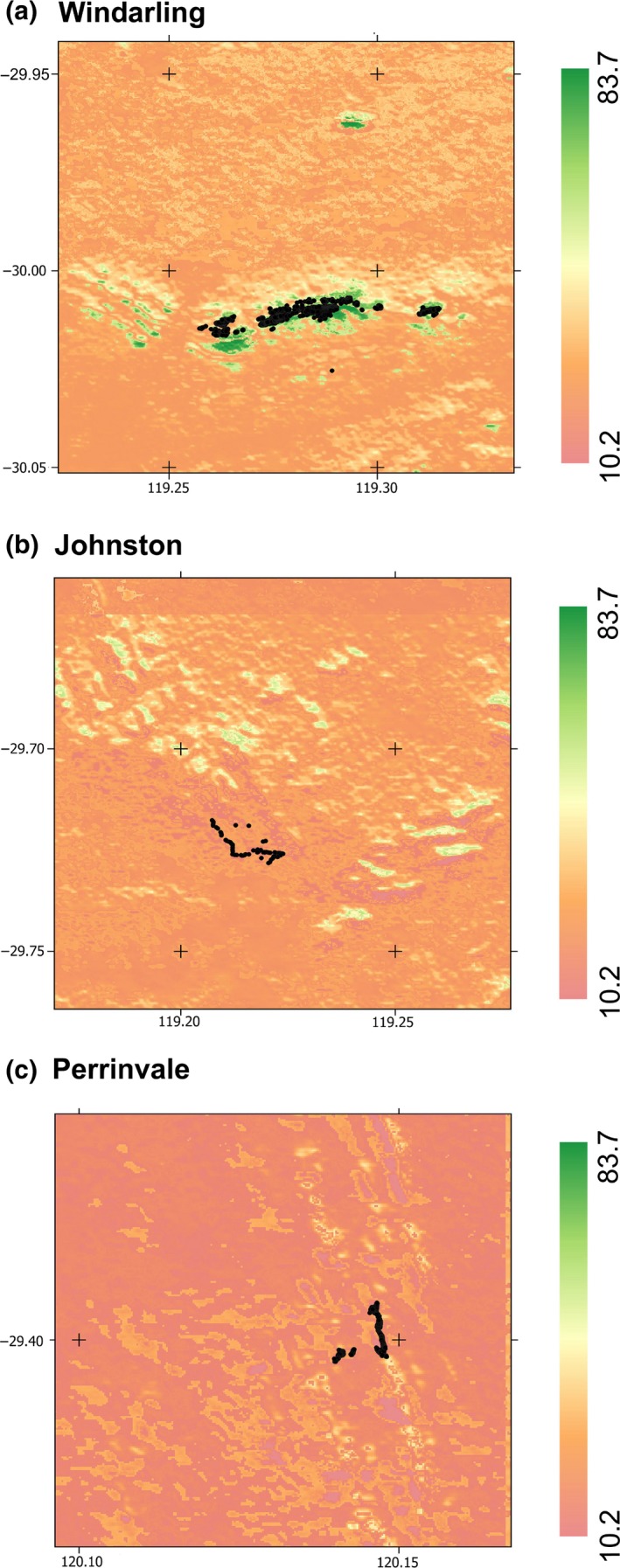
Projected species distribution of *R. brevis* likelihood of occurrence derived from Maxent model informed by microclimatic data describing patterns in air temperature, solar irradiance, soil water potential at a depth of 20 cm, and soil temperature at a depth of 10 cm during the austral winter at Windarling Range (a), Johnston Range (b), and Perrinvale Range (c). Increasing intensity of color (from red to green) indicates a high likelihood of occurrence

In order to compare the edaphic‐SDM with a more traditional, climatically informed model, we developed a climate‐SDM using the climatic layers estimated using the “micro_global” algorithm of the *NicheMapR* package (Kearney, 2016; Figure 5). We then randomly selected 3,000 points within these three landscapes and compared the projected habitat suitability estimated using the climate‐SDM with that estimated using the edaphic‐SDM using a paired *t* test where points that did not coincide with both projection areas were excluded.

## 3 RESULTS

### 3.1 Species distribution modeling

The final edaphic‐SDM of *R. brevis* obtained a high area under the curve (AUC = 0.95) within the training area. Potentially ranging between 0 and 1, an AUC value of 0.5 indicates that the model habitat selection is equivalently capable of differentiating presence and absence as a random selection, and a model is considered to represent a plausible habitat selection if the AUC is greater than 0.7 (Pearce & Ferrier, 2000). The MaxEnt response curves (Appendices S1 and S2) also suggest that the modeling captured a broad niche space. The average likelihood of occurrence at known locations of *R. brevis* was 72% (range = 0% to 93%). Over a quarter of known locations (encompassing 3,455 individuals) were modeled to have greater than 80% likelihood of occurrence, and two thirds of known locations (8,590 individuals) were modeled to have greater than 70% likelihood of occurrence. Only 492 known individuals were modeled as having less than 50% likelihood of occurrence. The final constrained edaphic‐SDM was constructed around soil depth, soil bulk density, elevation, aspect, sand, and silt content. The most powerful microhabitat factor determining the distribution of *R. brevis* was silt content (43%), followed by soil depth (24%), and soil bulk density (18%; Table 1). *Ricinocarpos brevis* was modeled to occur on shallow, low bulk density (i.e., well‐draining) soils that tend to occur high in the landscape.

**Table 1:** Relative contributions of the environmental variables to the final *Ricinocarpos brevis* MaxEnt distribution model

Variable	Percent contribution (%)	Permutation importance (%)
Edaphic model		
Silt content (%)	43.4	2.4
Soil depth (m)	24.2	36.8
Soil bulk density (g/cm^3^)	18.0	24.6
Elevation (m)	11.3	30.1
Aspect (°)	1.8	2.0
Sand content (%)	1.2	4.2
Microclimatic model		
Average air temperature	79.5	67.0
Soil temperature	20.1	30.9
Soil water potential	0.4	2.1

Note

Percent contribution is determined by the change in regularization at each iteration. Permutation importance is determined as the percentage normalized change in the area under the curve as the value is randomly permuted. The latter indicates the reliability of the factors in their contribution to the model.

### 3.2 Species distribution projections

Projection of the final model to encompass the three areas of interest (Figure 4) indicated that the intervening area between the known populations was largely unsuitable for *R. brevis*. Nevertheless, there are landscape features, especially between the Windarling and Johnston Ranges, that have high predicted likelihood (i.e., >80%) of supporting populations, but from which no populations are currently known. When interrogated for patterns of novelty, the majority of the broader projection landscape represented similar covariate space to the three training areas (Figure 4b). Where differences did occur, they were typically Type I differences, indicating that part of the landscape exceeded the univariate patterns of the training set, but that the covariance structures were stable (Mesgaran et al., 2014), which typically occurred at the highest elevations. The projection space at these higher elevations was characterized by soil and geomorphology that are not present within the training areas on the ridges where *R. brevis* is known to occur. As such, while much of the projection area was generated based on interpolation, the projections generated at the highest parts of the landscape were based on extrapolation rather than interpolation and should be treated with some caution. The total range of elevational change in our project area is approximately 200 m, and the extrapolations, by definition, encompass more than this.

### 3.3 Ecophysiological correlates of distribution

The soil temperatures over which the likelihood of occurrence was correlated for the austral winter, ranged from 18.6°C at 100 cm depth to 26.0°C at the soil surface, while the soil water potentials ranged from −0.05 kpa at 100 cm depth to −138.1 kpa at the soil surface (Table 2). There was a significant linear relationship between the modeled likelihood of occurrence of *R. brevis* and the microclimatic variables modeled for the training regions at Windarling, Perrinvale, and Johnston Ranges (*F*_11, 1988_ = 5.85, *p* < .0001). Model averaging resulted in three equally parsimonious models (Table 3), indicating strong support that the positive relationship between likelihood of occurrence and microclimatic projections was driven by soil water potential at 0 cm depth (*F*_1, 1995_ = 19.4, *p* < .0001), soil water potential at 0.2 m depth (*F*_1, 1995_ = 11.9, *p* < .0001), and soil temperature at 1.0 m depth (*F*_1, 1995_ = 15.4, *p* < .0001).

**Table 2:** Summary details of the microclimatic conditions calculated at 2000 random locations across the project area

		Surface	20 cm	100 cm
Air temperature (°C)	min	14.61		
	max	18.12		
	avg	16.46 ± 0.019		
Solar Radiance (lumens)	min	176.41		
	max	198.80		
	avg	189.58 ± 0.116		
Soil temperature (°C)	min	21.61	20.75	18.65
	max	25.99	25.05	22.69
	avg	23.94 ± 0.023	23.03 ± 0.023	20.80 ± 0.022
Soil water potential (kPa)	min	−91.24	−0.63	−0.05
	max	−138.16	−4.82	−0.08
	avg	−116.59 ± 0.233	−2.00 ± 0.020	−0.08 ± 0.000

Note

Air temperature and solar radiance were calculated once at each location, while soil temperatures and soil water potentials were calculated at three depths.

**Table 3:** AIC comparisons of microclimatic correlates of likelihood of occurrence in *R. brevis*

Model	*k*	Δ AICc
likelihood ~ SWP(0) + SWP(20) + 1	4	0.00
likelihood ~ temp(100) + SWP(0) + SWP(20) + temp(100):SWP(0) + 1	6	0.32
likelihood ~ temp(air) + SWP(0) + SWP(20) + 1	5	0.81

Note

Soil water pressure at different depths in centimetres is indicated by SWP(), while soil temperatures at different depths in centimetres is indicated by temp().

Projection of the climate‐SDM across the three areas of interest (Figure 5) indicated high habitat suitability that largely coincided with the known locations of the three populations. The known locations occurred in areas with 63.4 ± 0.20% microclimatic suitability. Consistent with our ecophysiological interrogation of the edaphic‐SDM, average temperature made the strongest contribution to the climate‐SDM, followed by soil temperature (Table 1). At the 3,000 randomly selected locations across the climate‐SDM, there was significant divergence in the estimated habitat suitability between the two models (*t*_2808_ = 64.6, *p* < .0001). The absolute mean difference in habitat suitability between the two models was 21.4 ± 3.11%.

## 4 DISCUSSION

Our over‐arching aim was to develop a standardized procedure by which SDMs could be constructed at a resolution and extent high enough to be practically informative for conservation management programs focussing on species that have highly localized distributions. By way of case study, we identified locations most suited to *R. brevis*, modeled upon correlations of geomorphology and soil type in its known locations. The result of a standardized procedure was targeted toward a mechanistic interpretation of the microclimatic factors most limiting the distribution of SRE taxa in order to suggest what future research is required to understand, and ameliorate, limitations in the management and restoration of this species.

### 4.1 Model extrapolations

The models described the distribution of *R. brevis* populations using freely available data on Australian soils, elevation and aspect and were prepared over a period of a single week on standard personal computers. We did not explicitly seek to exclude microclimate variables from our modeling, and the process that we advocate could, with appropriate computational power, develop highly resolved climate layers. However, this level of computing power can be prohibitive to uptake of these modeling approaches by many stakeholders, and we sought a way to resolve a suitable, high‐resolution SDM using freely available data layers on standard computing hardware that could also inform microclimatic analysis on a subset of locations. Using this edaphic‐SDM approach, *R. brevis* populations were modeled to occur on shallow, well‐drained soils. Although elevation emerged as an important factor driving the distribution of *R. brevis*, this is probably only because BIF is relatively resistant to weathering, and shallow, well‐drained soils on BIF mainly occur higher on the landform (Gibson et al., 2010), rather than because elevation has any intrinsic importance to the species: that is, that elevation is a proxy for BIF. We conclude this on the basis that the very low elevations in question, between 300 and 650 m above sea level (Figure 3) are unlikely to result in altitudinal climate shifts, as these localized ranges are generally no more than ~200 m above the surrounding plains. The exception to this, at least on face value, is the Johnston Range population, which persists on deeper soils, partly independent of BIF (Department of Environment & Conservation, 2011). The soil depth layers that we used here, however, characterize this region as a mosaic of shallow soils, between 30 and 50 cm deep, even though the elevation model (Gallant et al., 2011) does not indicate obvious geomorphological features that separately characterize the other two populations.

While relatively little land area outside of the existing populations was predicted as habitat highly likely to support *R. brevis*, there were nevertheless five distinct uplifted BIF regions of approximately 1,500 km^2^ that had a high modeled likelihood of supporting *R. brevis* (Figure 4a). Given that a number of very highly suitable habitats occur in the broader region that are uninhabited by this species, it seems likely that stochastic local extinctions have played a substantial role in the distribution of *R. brevis*, as with other flora species associated with BIF ranges in the region (Byrne et al., 2018). In this regard, the initial species distribution modeling approach based entirely on edaphic features, independent of the usual climatological data that informs correlative SDMs (e.g., Hijmans et al., 2005; Kearney, Isaac, et al., 2014) appears entirely suitable for the production of insightful high‐resolution SDMs for SREs. Furthermore, the SDM produced here was trained by freely available data sets that span all of continental Australia, such that any other models produced using these data should be directly comparable and equally interpretable to this one.

The climate‐SDM was much less accurate in discriminating between pockets of highly suitable habitat and less suitable habitat. Partly, this probably reflects the microclimates that we used to train the model, being the conditions in the coolest and wettest times of the year, when *R. brevis* is more likely to germinate and recruit (Turner et al., 2018). While these conditions provide one distributional constraint through recruitment, they probably do not provide the most powerful limitation to persistence, which is likely to happen either when conditions exceed a tolerance threshold (Buckley et al., 2010), or when sub‐lethally challenging conditions restrict physiological performance to the extent that that prevent growth and reproduction (Evans, Diamond, & Kelly, 2015). It may also be that, since we were restricted to developing microclimate layers for three relatively small landscapes, the pseudoabsences used by MaxEnt to estimate unsuitable conditions did not encapsulate a broad enough extent to strongly discriminate between suitable and unsuitable habitat. Nevertheless, similar spatial patterns emerged, and the same vacant pockets of potentially suitable habitat were identified as we found with the edaphic‐SDM. In light of the aims of this study, which were to test whether habitat suitability could be modeled reliably using only edaphic constraints (sensu Velazco, Galvão, Villalobos, and Marco Júnior, 2017), the climate‐SDM certainly reinforces the patterns observed in the edaphic‐SDM. Furthermore, the intensive calculations required to provide appropriate microclimate data suggest that the practical return on investment is greater in training a model of this type using edaphic data alone, rather than calculating microclimatic layers.

As a caveat on the interpretation of this model, the training areas were identified using one of the finest‐scale environmental classification schemes published (Mackey et al., 2008), but the known populations are tightly clustered even within these areas (see Figure 2). Given the propensity for MaxEnt and other correlative processes to overfit (Buckley et al., 2010), it may be that the niche space generated by even these relatively localized training areas is too broad to result in accurate outputs. Also, all models are heavily dependent on the consistency and quality of the data on which they are trained and projected. The freely available data sets used were derived from large computing efforts unifying data collected via remote sensing, which sometimes incorporate artefacts indicating varying precision across tile boundaries, and this is evident in the projection data here (Figure 3). Although we acknowledge that it may reduce accuracy of this layer for prediction, we have no way to quantify the spatial bias it implies, and no capacity to correct, nor strongly interrogate the source data. In this regard, the edaphic‐SDM of *R. brevis* produced is nevertheless plausible, though further interpretation is required to ascertain whether or not the resulting correlations reflect the mechanisms underlying the distribution (Buckley et al., 2010; Meynard & Quinn, 2007).

### 4.2 Ecophysiological interpretations

Most SDMs are trained using climatic data, but over small distributions, such as those relating to individual management projects, SREs or mining tenements, the available global climate projections are too coarse a resolution to provide meaningful insight into microclimatic variability (Tomlinson, Webber, Bradshaw, Dixon, & Renton, 2017). Indeed, there is growing recognition that plant distributions can be heavily influenced by below‐ground microclimatic shifts driven by soil types within otherwise homogenous climatic regions (Velazco et al., 2017). By specifically choosing to develop the edaphic‐SDM based on the geomorphological and soil features that inform the microclim data set (Kearney, Isaac, et al., 2014), we were able to calculate microclim estimates across the projection area, including below‐ground microclimate. We feel that our proposed approach is advantageous to the study of short‐range endemism due to its breadth of applicability. One of the challenges facing studies of SRE flora, especially in highly biodiverse landscapes such as the BIF outcrops of south‐western Western Australia (Gibson, Prober, Meissner, & Leeuwen, 2017; Gibson et al., 2010), Table Mountain National Park in South Africa (Helme & Trinder‐Smith, 2006), or archipelagos such as New Caledonia (Gâteblé et al., 2019; Kier et al., 2009) is that there may be a large number of SRE species, but they are usually either entirely unique, or a unique clade of closely related species within a taxon. In either case, it is very difficult to untangle the paired effects of ecology and phylogeny (Felsenstein, 1985). The approach that we have used here is dependent upon a standardized set of spatial data that can be applied at the same resolution to any endemic species in Australia. Furthermore, where similar data sets are available globally (Hengl et al., 2014), the *micro_global* algorithm can be used to calculate down‐scaled microclimate estimates using a standardized approach anywhere in the world. As such, this allows direct comparison of the drivers of short‐range endemism across similar habitats with markedly different flora throughout the world, such as the endemic flora of granitic inselbergs in Africa, Asia, South America, and Australia (Porembski, Seine, & Barthlott, 1997). The limitation to such a comparative study of the ecophysiological drivers of short‐range endemism in plants is that the global soil grids are currently available at a resolution of 1 km (Hengl et al., 2014), or roughly 30 arc‐sec, which is the same resolution as most of the freely available digital climate layers which we have already demonstrated are at too coarse a resolution to be applied to SRE flora (Figure 2). Currently, therefore, we can only pursue the ecophysiological interpretations that we have gleaned for our case study species.

The most critical microclimatic factor contributing to the distribution of *R. brevis* was soil water potential at biologically relevant depths (i.e., 20 cm to 1 m). Indeed, our models suggest that *R. brevis* may be restricted to comparatively wetter soil conditions in an otherwise arid environment, indicating that *R. brevis* may be refugial in highly localized milder and wetter niches. Recent research showed high susceptibility of seed germination to water stress, with requirements of above −90 kpa soil water potential for optimal germination over a relatively long period of time (Turner et al., 2018), is highly consistent with the modeled expectations. We expect these processes to rely on frequent and sustained moisture availability, although these events are likely to be irregular and unpredictable (Elliott, Lewandrowski, Miller, Barrett, & Turner, 2019; Miller, Symonds, & Barrett, 2019). Given the hot semi‐arid environment where *R. brevis* is found, this may relate to a complex set of plant traits regulating water loss and water uptake (Lambers et al., 2008). Understanding these mechanisms should be a component of any future species‐specific ecophysiological studies on this species with our model identifying several potentially insightful lines of scientific enquiry.

### 4.3 Future research and management directions

In light of the anthropogenic disturbances to this species, and the highly restricted locations of the known populations, there are several conservation opportunities to minimize the risk of extinction and improve species recovery identified by our SDMs. The simplest to justify is the supplementation of existing populations via direct seeding or the planting of nursery produced seedlings (Coates et al., 2014). Even at the known populations, the modeling suggests that not all suitable habitat is filled (Figure 4). The identification of vacant niche space at known populations provides an apparent opportunity to supplement and increase these populations to offset losses due to anthropogenic disturbance. A more complex possibility raised by the model outcomes is the establishment of a population in a landscape bearing a conservation covenant. There are locations currently not known to support *R. brevis,* but with high modeled suitability within the proposed Mount Manning conservation reserve at the Die Hardy Ranges and could be a suitable location for the establishment of an insurance population of the species, if such was required for conservation management and successful translocation approaches into the natural environment were already known.

Assisted colonization is a philosophy originally proposed for moving species threatened by changing climates (McLachlan, Hellmann, & Schwartz, 2007; Webber, Scott, & Didham, 2011) that is gaining traction rapidly as a conservation tool (Sgrò, Lowe, & Hoffmann, 2011). Correlative modeling approaches such as MaxEnt are often used to guide these enterprises (Ferrarini et al., 2016; Regan et al., 2012). Translocation to establish insurance populations is also a common conservation practice for rare or range‐restricted animals and plants (Griffith, Scott, Carpenter, & Reed, 1989; Morris et al., 2015; Silcock et al., 2019). The challenge in establishing insurance populations for SRE plants following distribution modeling has generally been the difficulty in constructing suitable models at high resolution. The workflow that we advocate here produces SDMs that are not specifically trained by climate data, and so cannot be readily extrapolated into future climate scenarios. This limits their capacity to identify targets for climate‐sensitive assisted colonization. For guiding the establishment of insurance populations under presumed stable climates, however, our workflow would be generally valuable. An argument could be made that ignoring global climate change in any conservation venture is naive, and ultimately self‐defeating, but Harrison and Noss (2017) note that the areas of highest short‐range endemism tend to also be associated with long‐term climatic stability, so as long as the insurance populations are not proposed to be established outside the climate region of other known populations the modelled locations should remain valuable to guide conservation efforts. However, with current global climate change proceeding at rates and magnitudes that eclipse all previous known periods of changing global climate in more recent times, this may no longer be a safe assumption.

While the MaxEnt model appears convincing, we must be measured in our interpretation and remain mindful that the modeling process is correlative. MaxEnt simply finds environmental correlates of population boundaries, and not the underlying biological or ecological drivers that regulate species occurrence. Interpretation of these projections, including interpretation of their plausibility, is subjective. The ultra‐high‐resolution projections reported here suggest some ecophysiological interpretations that are more strongly substantiated than other high‐resolution MaxEnt models, because the association between the environmental data and the resulting microclimatic is empirical, rather than implicit, but the actual ecophysiological mechanisms that restrict *R. brevis* to these habitats remains unresolved. Further ecophysiological studies provide the raw performance functions around which more flexible and insightful niche envelope modeling (Kearney, 2006) can re‐interpret the same high‐resolution training data presented here and mark the next step in the evolution of a standardized and comparable modeling approach to support the conservation of SRE flora.

## CONFLICT OF INTEREST

None declared.

## AUTHOR CONTRIBUTIONS

S. Tomlinson developed the modeling approach with consultation by B. Miller. C. Elliott, W. Lewandrowski and S. Turner provided biological interpretation. All authors contributed to the management implications and writing the manuscript.

## Data Availability

Projection layers, microclimatic analysis and full model projections are available at: https://doi.org/10.5061/dryad.5mkkwh71s . Training data: There is an embargo on the publication of locations of individual plants under the conservation legislation protecting these species in Australia. Thus, locations cannot be made public.
