# Supplementary figures and images for: High‐resolution distribution modeling of a threatened short‐range endemic plant informed by edaphic factors
Source: Ecol Evol. 2019 Dec 15;10(2):763–77. doi: 10.1002/ece3.5933 (PMC6988535; doi:10.1002/ece3.5933)

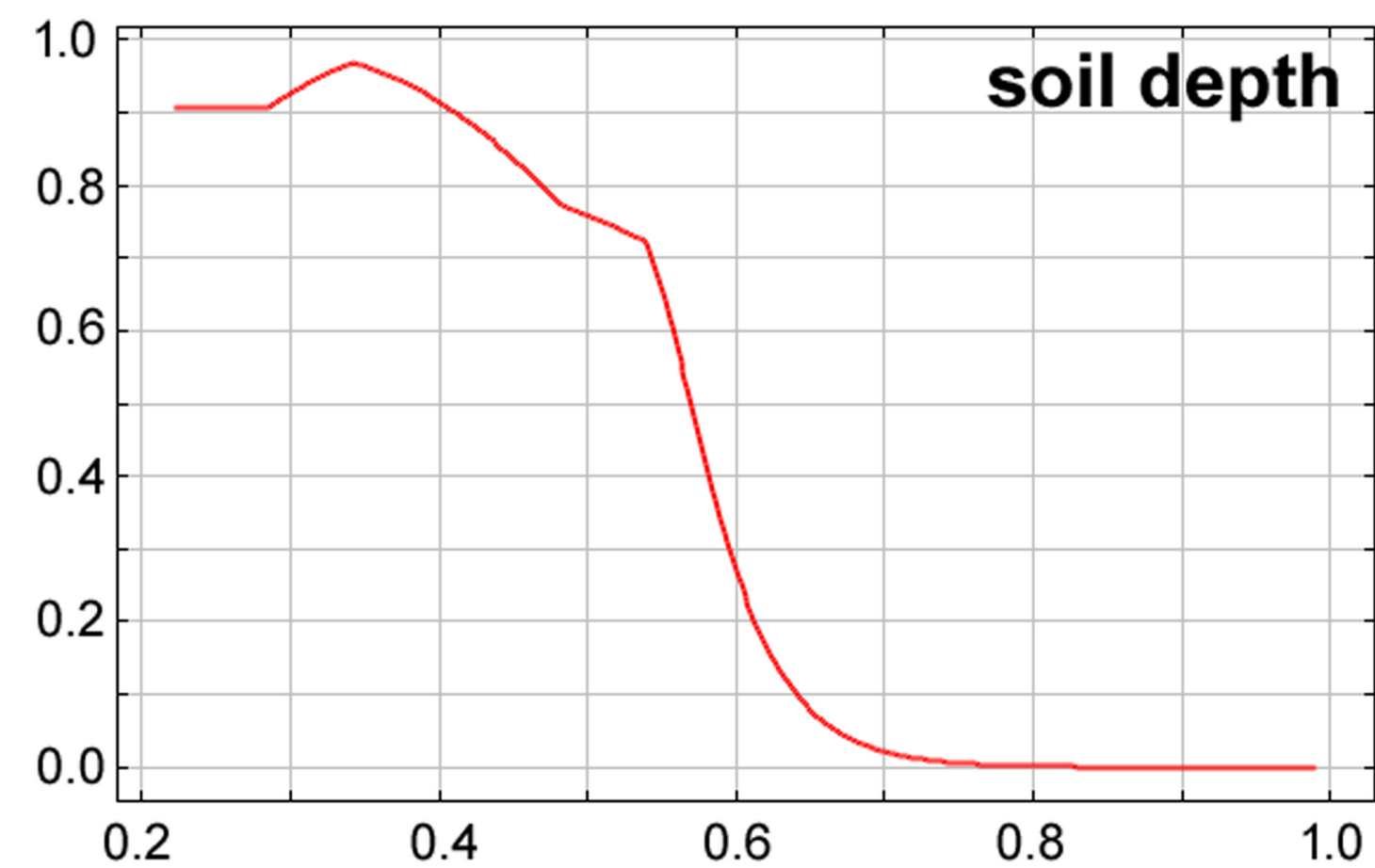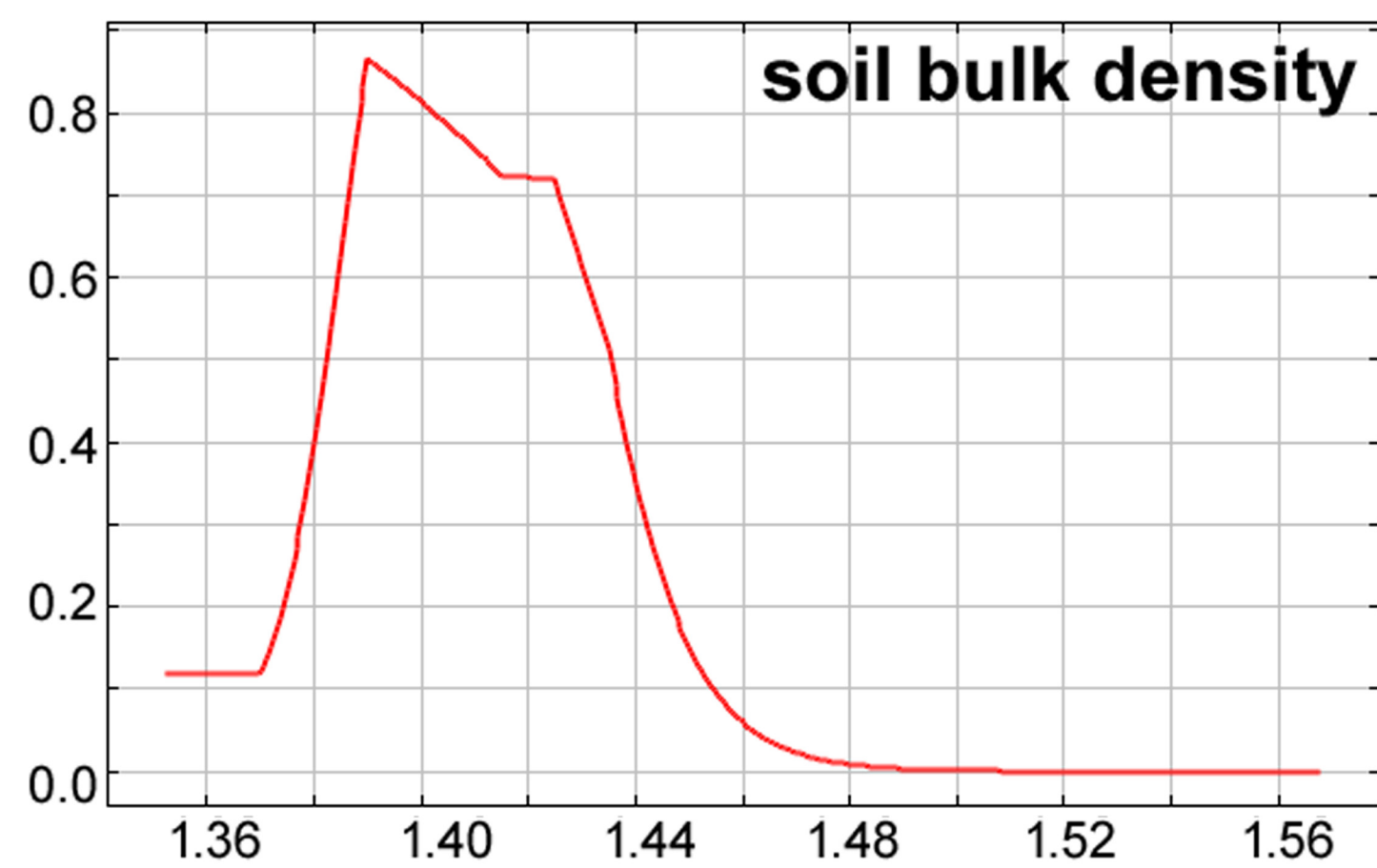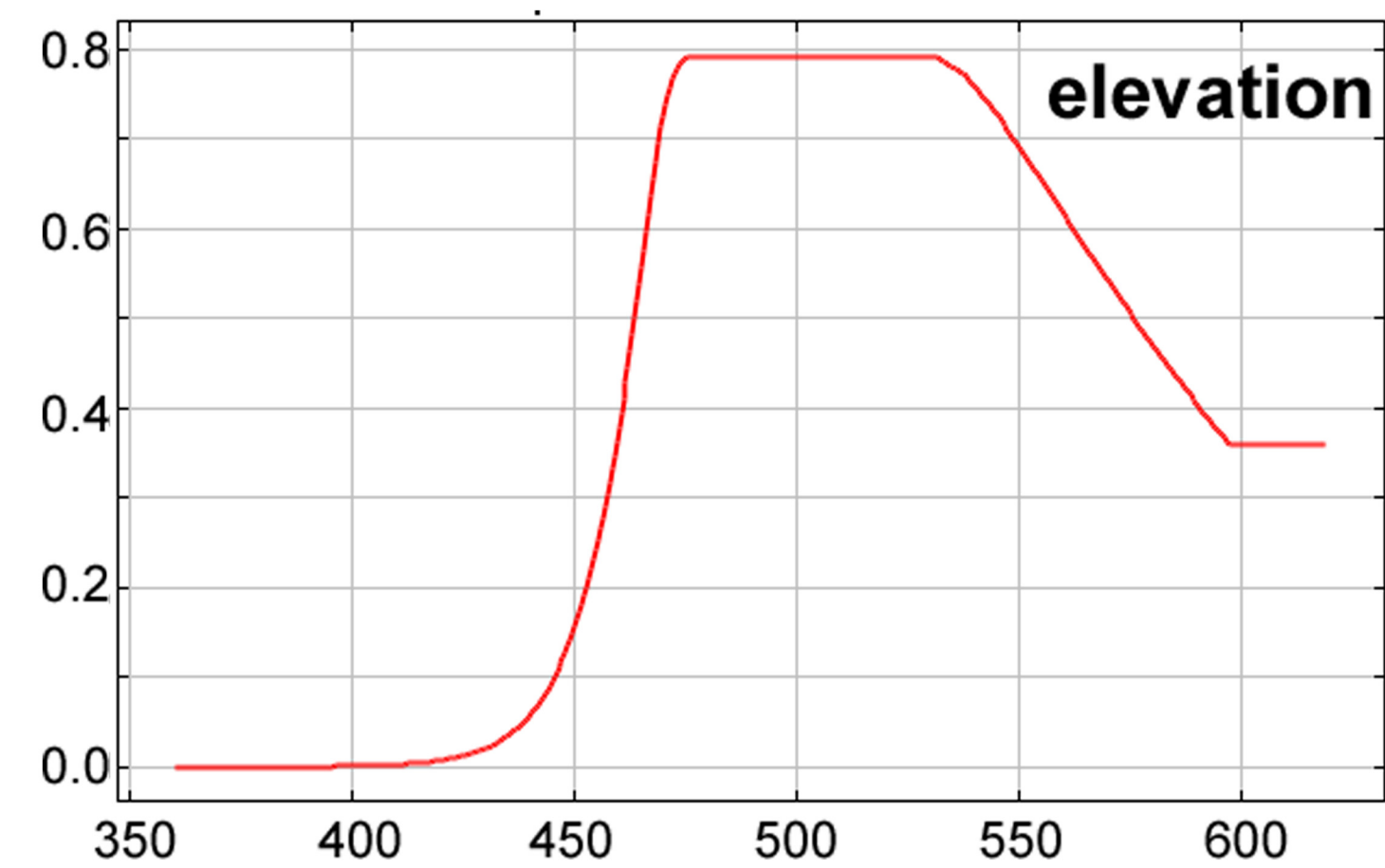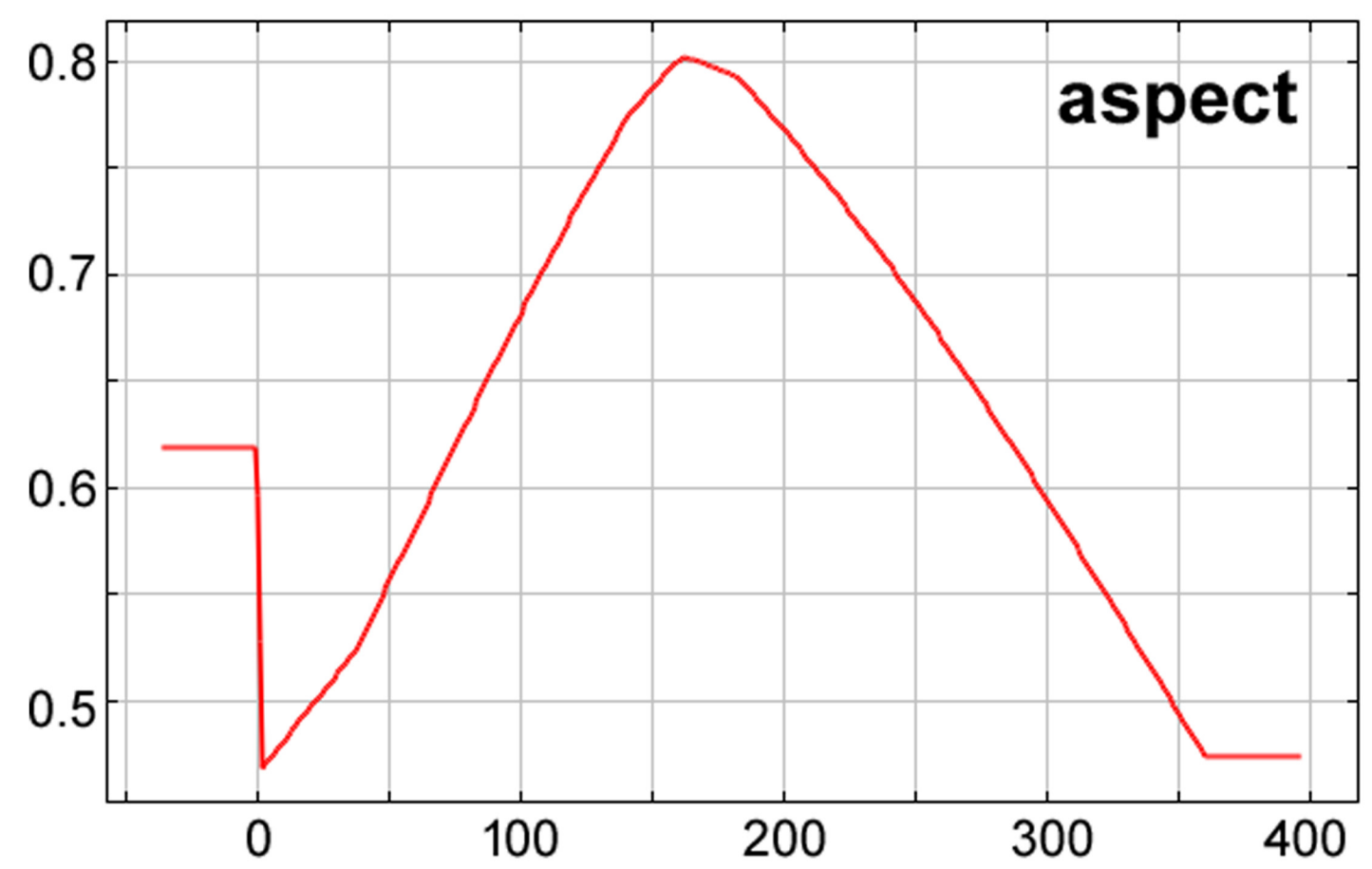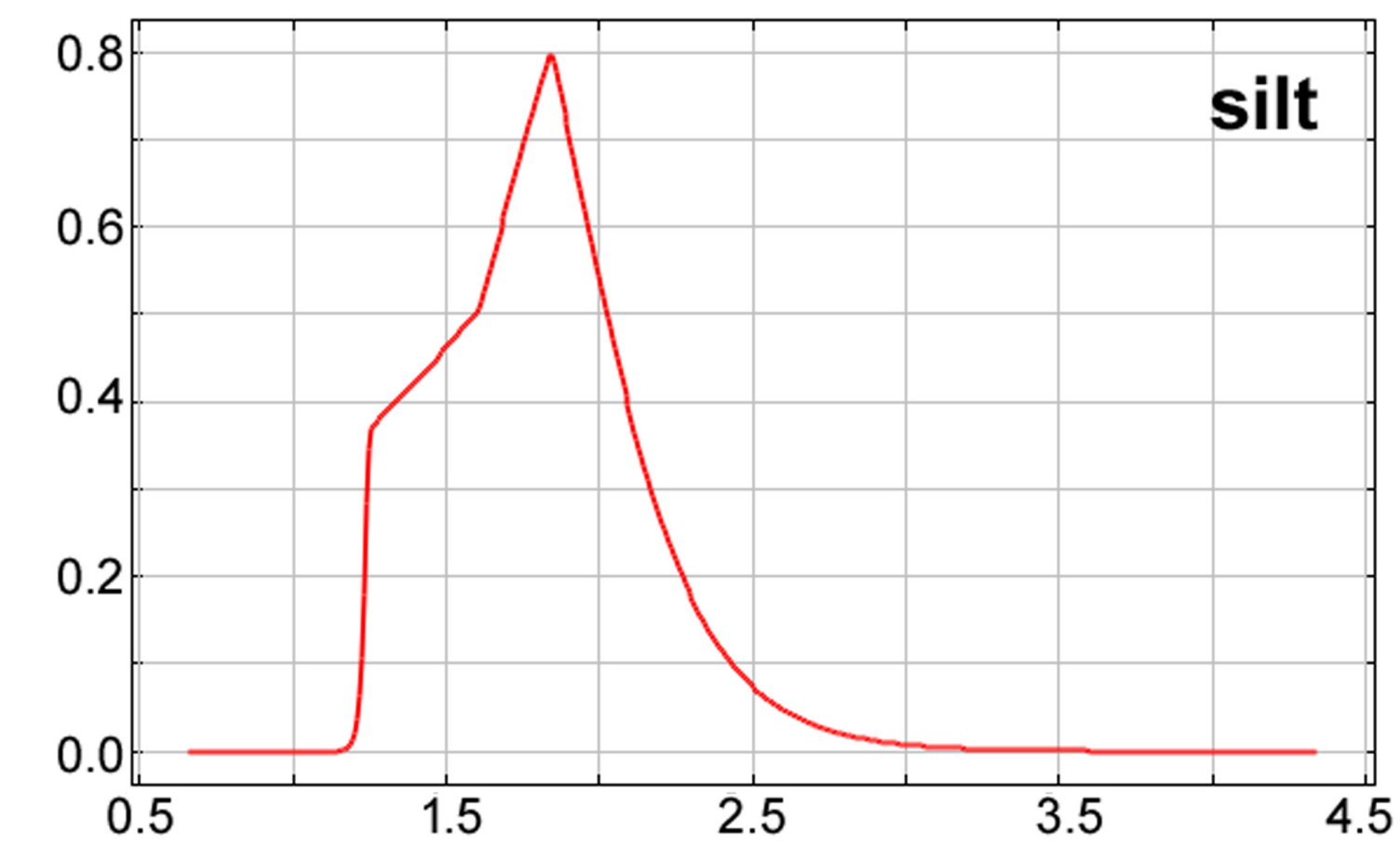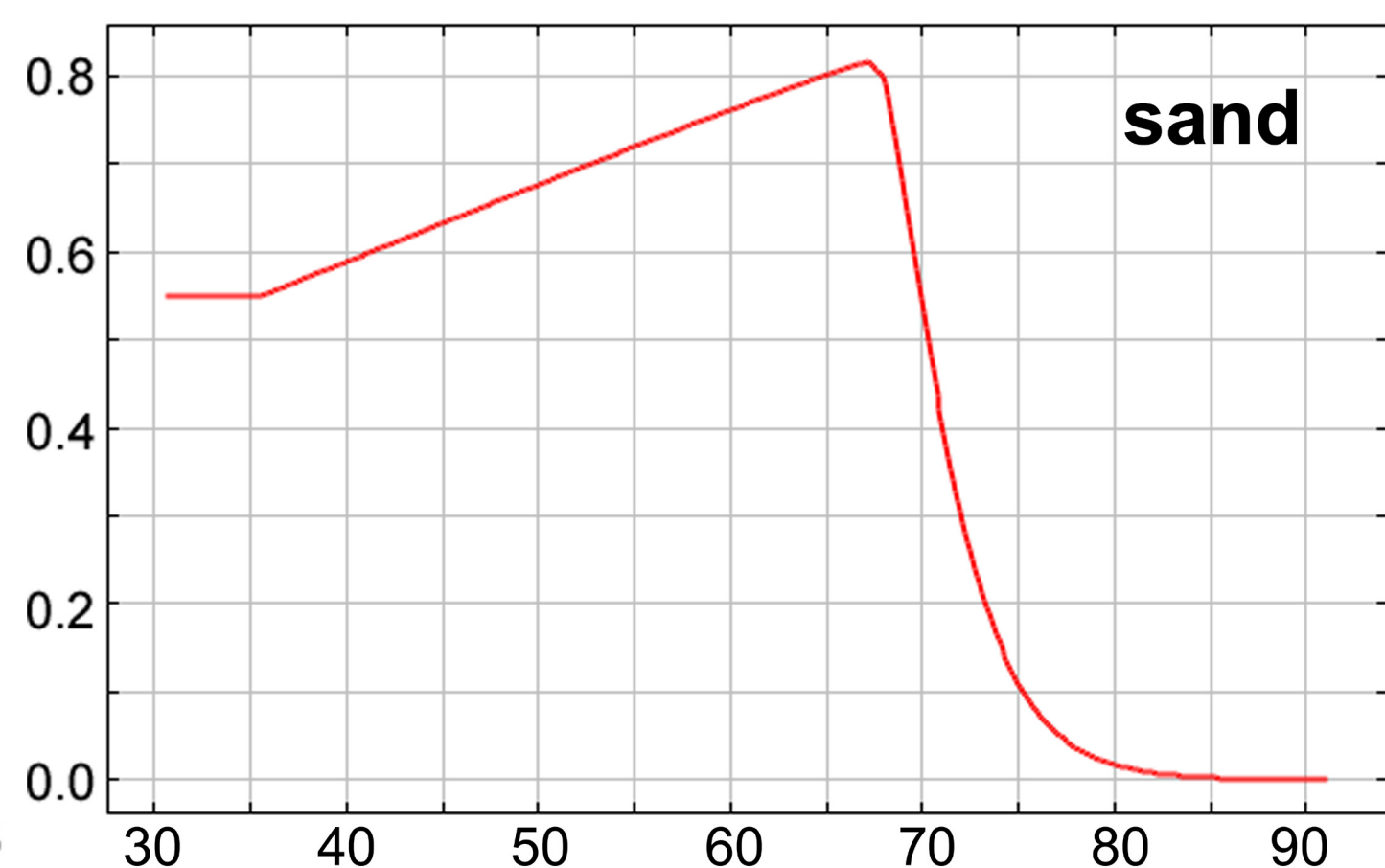

Supplement: Supplementary file 1 [file ECE3-10-763-s001.pdf]

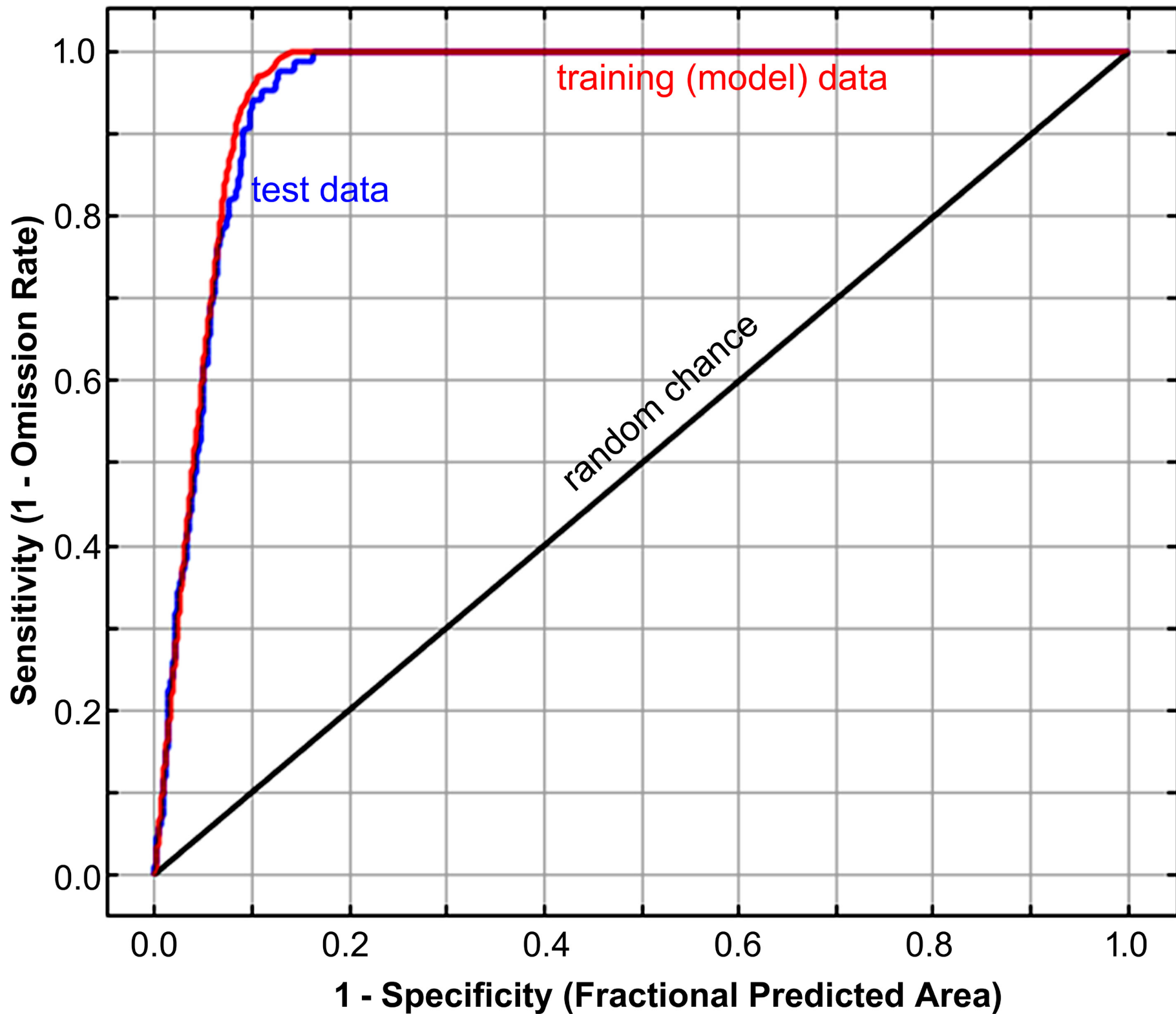

Supplement: Supplementary file 2 [file ECE3-10-763-s002.pdf]
