# Supplementary material for: High‐resolution distribution modeling of a threatened short‐range endemic plant informed by edaphic factors
Source: Ecol Evol. 2019 Dec 15;10(2):763–77. doi: 10.1002/ece3.5933 (PMC6988535; doi:10.1002/ece3.5933)
Supplement: Supplementary file 3 [file ECE3-10-763-s003.docx]

**Appendices: MaxEnt response curves and receiver operator curves**

**Appendix S1:** Marginal response curves of MaxEnt probability predictions to each distributional forcing factor, reflecting the dependence of predicted suitability both on the selected factor and on dependencies induced by correlations between the selected factor and other factors. The majority of forcing factors show low probability “shoulders” on each side of the distribution peak, suggesting broad enough backgrounds to generate modeling space.

**Appendix S2:** Receiver operating characteristic (ROC) curve generated by MaxEnt, where the true positive rate (Sensitivity) is plotted as a function of the false positive rate (100-Specificity). Essentially, the maximum accuracy possible is an area under the curve of 1.0, where all positive returns are true. If AUC of the model is 0.5 or less, it predicts distribution no more effectively than chance.
